# HelixGAN a deep-learning methodology for conditional *de novo* design of α-helix structures

**DOI:** 10.1093/bioinformatics/btad036

**Published:** 2023-01-18

**Authors:** Xuezhi Xie, Pedro A Valiente, Philip M Kim

**Affiliations:** Donnelly Centre for Cellular and Biomolecular Research, University of Toronto, Toronto, ON M5S 3E1, Canada; Department of Computer Science, University of Toronto, Toronto, ON M5S 3E1, Canada; Department of Computer Science, University of Toronto, Toronto, ON M5S 3E1, Canada; Donnelly Centre for Cellular and Biomolecular Research, University of Toronto, Toronto, ON M5S 3E1, Canada; Department of Computer Science, University of Toronto, Toronto, ON M5S 3E1, Canada; Department of Molecular Genetics, University of Toronto, Toronto, ON M5S 3E1, Canada

## Abstract

**Motivation:**

Protein and peptide engineering has become an essential field in biomedicine with therapeutics, diagnostics and synthetic biology applications. Helices are both abundant structural feature in proteins and comprise a major portion of bioactive peptides. Precise design of helices for binding or biological activity is still a challenging problem.

**Results:**

Here, we present HelixGAN, the first generative adversarial network method to generate *de novo* left-handed and right-handed alpha-helix structures from scratch at an atomic level. We developed a gradient-based search approach in latent space to optimize the generation of novel *α*-helical structures by matching the exact conformations of selected hotspot residues. The designed *α*-helical structures can bind specific targets or activate cellular receptors. There is a significant agreement between the helix structures generated with HelixGAN and PEP-FOLD, a well-known *de novo* approach for predicting peptide structures from amino acid sequences. HelixGAN outperformed RosettaDesign, and our previously developed structural similarity method to generate D-peptides matching a set of given hotspots in a known L-peptide. As proof of concept, we designed a novel D-GLP1_1 analog that matches the conformations of critical hotspots for the GLP1 function. MD simulations revealed a stable binding mode of the D-GLP1_1 analog coupled to the GLP1 receptor. This novel D-peptide analog is more stable than our previous D-GLP1 design along the MD simulations. We envision HelixGAN as a critical tool for designing novel bioactive peptides with specific properties in the early stages of drug discovery.

**Availability and implementation:**

https://github.com/xxiexuezhi/helix_gan.

**Supplementary information:**

Supplementary data are available at *Bioinformatics* online.

## 1 Introduction

Computational design holds enormous promise for protein engineering, and biomedicine with diagnostics, biosensors, therapeutics and synthetic biology applications. Successful deep-learning approaches like Alphafold2 have been developed for protein structure prediction (Jumper *et al.*, 2021). However, developing deep-learning methodologies for protein design remains challenging due to protein structure’s complexity and intricate functionality (Koga *et al.*, 2012; Rocklin *et al.*, 2017).

*α*-helix structures are the most commonly occurring secondary structure of proteins, imparting stability, representing 30% of the structure of the average globular protein (Lau and Dunn, 2018; Nick Pace and Martin Scholtz, 1998). It is reported that *α*-helical peptides take part in nearly 40% of the homodimeric and 26% of the heterodimeric protein–protein interfaces (Guharoy and Chakrabarti, 2007). However, native *α*-helical peptides are poor drug candidates because of their reduced conformational stability in the absence of the protein scaffold and their low resistance to proteolysis (Ali *et al.*, 2019).

Peptides consisting of D-amino acids have unique advantages, including low immunogenicity, manufacturing cost and high proteolytic stability (Kreil, 1997; Rabideau and Pentelute, 2015; Uppalapati *et al.*, 2016). Recently, we developed an in-house methodology for converting (L)-peptides to highly stable D-analogs after searching a mirror-image version of the protein data bank (D-PDB) (Garton *et al.*, 2018). D-peptide analogs capable of activating the GLP1 and PTH receptors (Garton *et al.*, 2018) and blocking the SARS-CoV-2 infection (Valiente *et al.*, 2021, 2022) have been designed using this pipeline. However, although the D-PDB offers a relatively extensive repertoire of native helical structures, it is still a tiny fraction of possible stable helices. One potential solution is to train deep generative models on the PDB to create novel helices.

Machine learning and more recently a deep-learning-based family of generative models called Generative Adversarial Networks (GANs) has gained significant traction in biology (Amimeur *et al.*, 2020; Gupta and Zou, 2018; Repecka *et al.*, 2021; Shuai *et al.*, 2021; Surana *et al.*, 2021) due to its capacity to identify patterns in vast, complicated datasets and produce synthetic data with desired features (Gulrajani *et al.*, 2017). Madani *et al.* developed ProGen, a 1.2 billion parameter language model trained on 280 million protein sequences. ProGen generates proteins that exhibit near-native structure energies, which likely imply functional viability (Madani *et al.*, 2020). ProteinGAN is another GAN recently developed that can ‘learn’ natural protein sequence diversity and generate functional protein sequences. ProteinGAN generated soluble protein sequences with malate dehydrogenase (MDH) catalytic activity using MDH as the training set (Repecka *et al.*, 2021). Kucera *et al.* (2022) developed ProteoGAN, a conditional GAN for protein sequence generation based on hierarchical labels from Gene Ontology.

Recently, Baker’s lab developed deep-learning approaches for scaffolding key functional sites in proteins without needing to prespecify the fold or secondary structure of the scaffold (Wang *et al.*, 2022). These authors also developed ProteinMPNN, a deep-learning-based protein sequence design method, to speed up the design process (Dauparas *et al.*, 2022). These methods were combined to create candidate immunogens, receptor traps, metalloproteins, enzymes and protein-binding proteins.

A popular GAN version that shortens the Earth Mover (Wasserstein) distance between real data and generated data is the Wasserstein GAN (WGAN) (Arjovsky *et al.*, 2017). The WGANs have been demonstrated to enormously improve the stability of the model training. Karimi *et al.* (2020) used a guided conditional WGAN to generate proteins with novel folds (Karimi *et al.*, 2020). Recently, WGANs models have been successfully applied for generating DNA and protein sequences with desired properties (Gupta and Zou, 2018; Repecka *et al.*, 2021). The feedback-loop architecture uses external function analyzers to gradually update the training data to produce DNA with desired properties, such as antimicrobial activity or secondary structure of encoded peptides (Gupta and Zou, 2018).

This manuscript presents an alpha-helix generative all-atom model using a WGAN. Our model uses a gradient search mechanism to generate *de novo* left- and right-handed helix structures matching hotspot residues in known helical structures. Significantly, the generated helical structures superimpose the template target hotpots with root mean square deviations (RMSDs) lower than 1.5 Å. HelixGAN outperformed our previously developed structural similarity method to generate D-peptides matching a set of given hotspots in a known L-peptide. We also designed a novel D-GLP1_1 analog that matches the conformations of critical hotspots for the GLP1 function as proof of concept. This novel D-peptide analog is significantly more stable than our previous D-GLP1 design along the molecular dynamics (MD) simulations.

## 2 Materials and methods

### 2.1 Database construction, encoding and metrics

The PDB stores over 100 000 protein structures and is a crucial resource for structural biologists. Our method captures all helices from the PDB to build our helical database. There are around 3 million helical structures in our database. The database was further divided into training and test datasets. We filtered the test dataset to ensure this subset is no more than 42.8% sequence identity compared with any data in the training set. Three hotspot residues were randomly selected on the test dataset to test our model performance. We prepared 3118 test samples containing three L-type hotspots. Then, we randomly chose half of them (1559 test samples) for testing the model performance to generate D-helical or L-helical structures.

We developed an encoding method where the helix structures are represented as the combined vectors of the sequence and structural features. The sequence is utilized as one-hot vectors and the structure is encoded by angle information (phi, psi, omega, bond angles for the main chain, and five chi angles for the side chain) (Supplementary Fig. S1). The mirror conversion is also applied as an optional step for GAN-generated helices to convert generated L-peptides into D-peptides since our motivation is to solve the data space limitation of D-analogs. We used REF2015, a popular physics-based energy score function implemented in the Rosetta program, to assess the quality of generated helical structures (Alford *et al.*, 2017).

### 2.2 HelixGAN model

HelixGAN utilized the WGAN architecture with gradient penalty. The WGAN is a variant of the GAN, which minimizes the Earth Mover (Wasserstein) distance between the distribution of real data and the distribution of generated data (Arjovsky *et al.*, 2017). A gradient penalty is imposed for gradients above one regarding the D of WGAN to maintain a Lipshitz constraint (Garton *et al.*, 2018). WGANs have been demonstrated empirically helpful in stabilizing the GANs’ training process. The flowchart of the model training is shown in Supplementary Figure S2. The model contains a generator (G) module, to generate data from latent space, and a discriminator (D), to distinguish whether the input data are real or not. Both modules are largely composed of ConvBlocks, which include Relu activation and Conv1d layers. When sampling from the generator, the argmax of the probability distribution is taken to output a single amino acid at each position with other internal coordinate features to construct the structure. In addition, the mirror transformation methodology can be applied to convert the generated L-peptides into D-peptide structures.

### 2.3 Latent space optimization search using structural constraints

We developed a gradient-based search mechanism in latent space to optimize the search of the desired structure given a set of specific target hotspots. Our optimization problem is to minimize the structural differences between the target hotpots and selected residues in the valid generated data. To do that, we first ensure the residues match the target hotspot residues following the hotspot matching rule defined previously by our group. The gradient search was implemented by calculating the cross-entropy loss between the generated amino acids and the targets to facilitate the search. After matching residues, we would calculate the structural differences using RMSD to find the ideal generated data that mimics the set of hotspots residues within an alpha-helical peptide structure. We began our searching process in multiple starting points of the latent space to improve the performance by preventing the system being trapped into local minima.

### 2.4 MD simulations

All the initial structures and topology files for the MD simulations of the GLP1 receptor (GLP1R) in complex with different peptides embedded into a POPC:PSM (1:1) bilayer were built using the membrane builder generator implemented in the CHARMM-GUI web server (Jo *et al.*, 2008; Lee *et al.*, 2016). The GROMACS software package (Abraham *et al.*, 2015) version 2019.3 was used to perform the MD simulations of the GLP1R+peptide complexes using the CHARMM36-m force field (Huang *et al.*, 2017) and the TIP3P water model (Jorgensen *et al.*, 1983). Two consecutive energy minimization schemes were used to initially relax the systems. The systems were then equilibrated in two sequential NVT ensemble simulations before being equilibrated in five successive NPT ensemble simulations at *p* = 1 bar, and *T* = 310 K. We gradually released the position restraints that had been applied to the protein-heavy atoms in both steps. Finally, the production NPT runs were performed for 200 ns for each system.

## 3 Results

### 3.1 HelixGAN a deep-learning method for the *de novo* design of *α*-helix structures

**Fig. 1.**
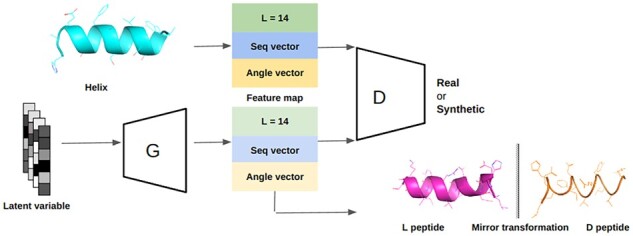
Flowchart of the *de novo* methodology for generating helical structures coined as HelixGAN. The Generator (G) network produces a full-atom helical structure from a random input latent vector, while the Discriminator (D) network scores it by contrasting it with natural helices. The feature map shows the encoded information to generate the full-atom structures, including the sequence (seq) and the structural information (angle) vectors, respectively. Both vectors share a length of 14 amino acids (*L* = 14). The generator network generates full-atom helices that eventually resemble real helices to trick the discriminator. This model directly generates L-helices since the training data were L-peptides. The model can also generate D-helical peptides by transforming the generated L-helices into D-peptides using a mirror transformation

Here, we presented HelixGAN, a deep-learning method for designing helical structures from scratch. HelixGAN generates novel full-atom helices using GANs. HelixGAN learns hidden patterns from sequences and structural features by combining a WGAN architecture with customized temporal convolutional networks. Because the training data were L-peptides, this model generates L-helices directly. The model can also generate D-helical peptides by mirror-transforming the generated L-helices into D-peptides (Fig. 1).

**Fig. 2.**
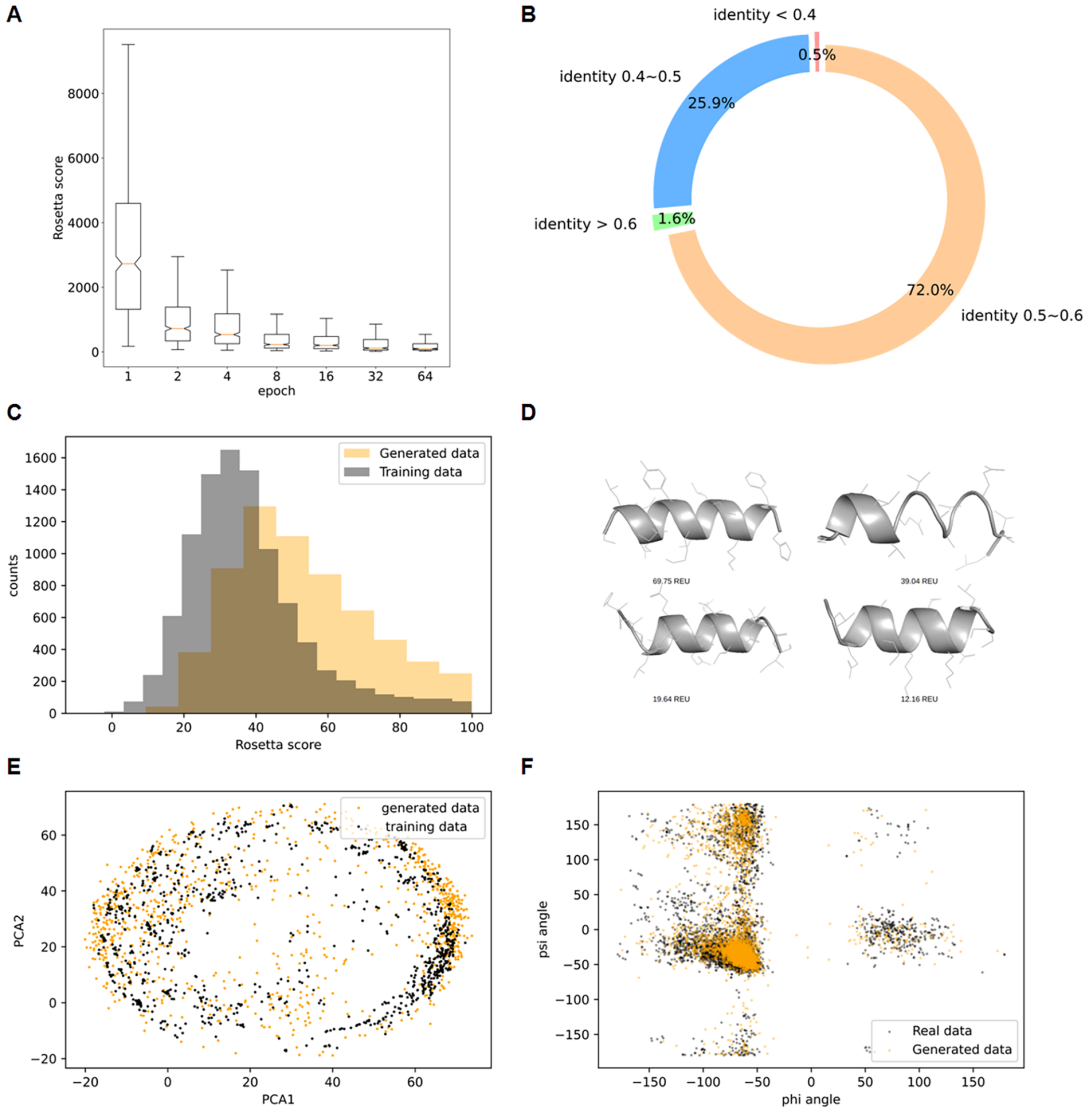
Assessment of the novel helices generated with HelixGAN. (**A**) Rosetta score distribution of 200 generated full-atom L-helices using fixed noises at different training epochs of HelixGAN. (**B**) Sequence identity between 10 000 randomly selected helices to the nearest natural helices obtained from the training data. (**C**) Rosetta score distribution of the 10 000 randomly selected generated full-atom helices and the training data. We only selected helices with scores smaller than 100 REU. (**D**) 3D structure of generated full-atom helices with Rosetta scores smaller than 100 REU. (**E**) PCA over the CA atom coordinates of 1000 randomly selected generated full-atom helices and the training data. (**F**) Ramachandran plot of 1000 randomly selected generated full-atom helices and the training data

We generated synthetic helical structures of 14 amino acids using HelixGAN. We monitored the quality of the generated structures regarding 500 fixed samples from the generator during the training using the Rosetta scoring function. The helix structures with a Rosetta score below 100 REU were classified as reasonable helical structures. Remarkably, none of the initially generated structures was classified as reasonable helices before training, while 61.4% of the sampled sequences contained reasonable helical structures after training (Fig. 2A). We next generated L-helix peptides of different lengths (16 and 18 amino acids) to evaluate the model performance by increasing the helix size. We observed that HelixGAN performance decreases when we increase the length of the designs (Supplementary Fig. S3). We also implemented the Spectral GAN network (Miyato *et al.*, 2018) and generated 500 designs of L-helix peptides with this new model for comparison with WGAN architecture. The results showed that WGAN slightly outperformed Spectral GAN regarding the Rosetta energy scores (Supplementary Fig. S4).

Following that, we tested the structure’s quality by randomly sampling 10 000 helices from HelixGAN. Notably, most of the generated data (72%) have sequence identity with the training data between 50% and 60% (Fig. 2B). The generated data contain a similar range of physically reasonable helices as the training data (Fig. 2C and D). We evaluated the model’s performance by selecting a lower sequence filtering cut-off (35% instead of 42.8%). Of note, we observed a significant decrease in the quality of the helix structures generated with HelixGAN. We think that the model performance will decrease more if we reduce the sequence identity in the filtering step of our pipeline (Supplementary Fig. S5).

Principal component analysis showed that the generated and natural helix structures share a similar conformational space (Fig. 2E). The Ramachandran plot also showed a similar profile for the generated and training helix structures dataset (Fig. 2F). Notably, some residues within the generated and training datasets are distributed in beta-sheet regions. A careful analysis of our data indicated that most of these residues are terminal helix residues. Significantly, Gly and Pro were these populations’ most commonly found residues. The analysis of the chi angles of the generated and natural helices also showed a similar distribution between both datasets (Supplementary Fig. S6). Based on these analyses, we concluded that HelixGAN generated reasonable helix structures with a similar distribution as the natural helices.

To compare HelixGAN with other deep-learning-based protein sequence design methods, such as ProteinMPNN (Dauparas *et al.*, 2022) and ESM-inverse (Wang *et al.*, 2022), we generated 3000 random L-helices and calculated the sequence recovery rate using our training dataset. As expected, we observed an increase in the sequence recovery by decreasing the temperature (Supplementary Table S1). Notably, we got a significantly lower native sequence recovery to generate L-helical peptides with both methods (<20%) than the ones obtained by their authors to design sequences (51–52%) that fold into the desired structure. Given the low native sequence recovery, we did not further test both algorithms to generate new helices from scratch.

**Fig. 3.**
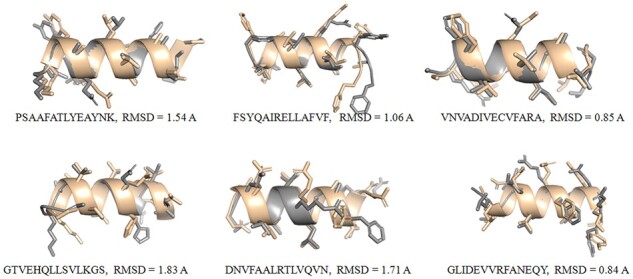
Structural superposition of the designed helices generated with HelixGAN and PEP-FOLD. In gray are highlighted the helical structures generated with HelixGAN while in light orange are displayed the helix structures generated with PEP-FOLD (A color version of this figure appears in the online version of this article)

Finally, we compared the structural similarity between the alpha right-handed helix structures generated with HelixGAN and PEP-FOLD (Lamiable *et al.*, 2016), a well-known *de novo* approach for predicting peptide structures from amino acid sequences. To compare both methodologies, we picked up a small random number of sequences. Notably, our results showed a good match between the structures generated with both methods for similar sequences. In all cases, we observed RMSD below 2.0 Å. In some test cases, the more significant differences observed are due to different rotamers sampled by both methods in the side chains of large residues (Fig. 3).

### 3.2 *De novo* design of peptides targeting constrained hotspots using HelixGAN

**Fig. 4.**
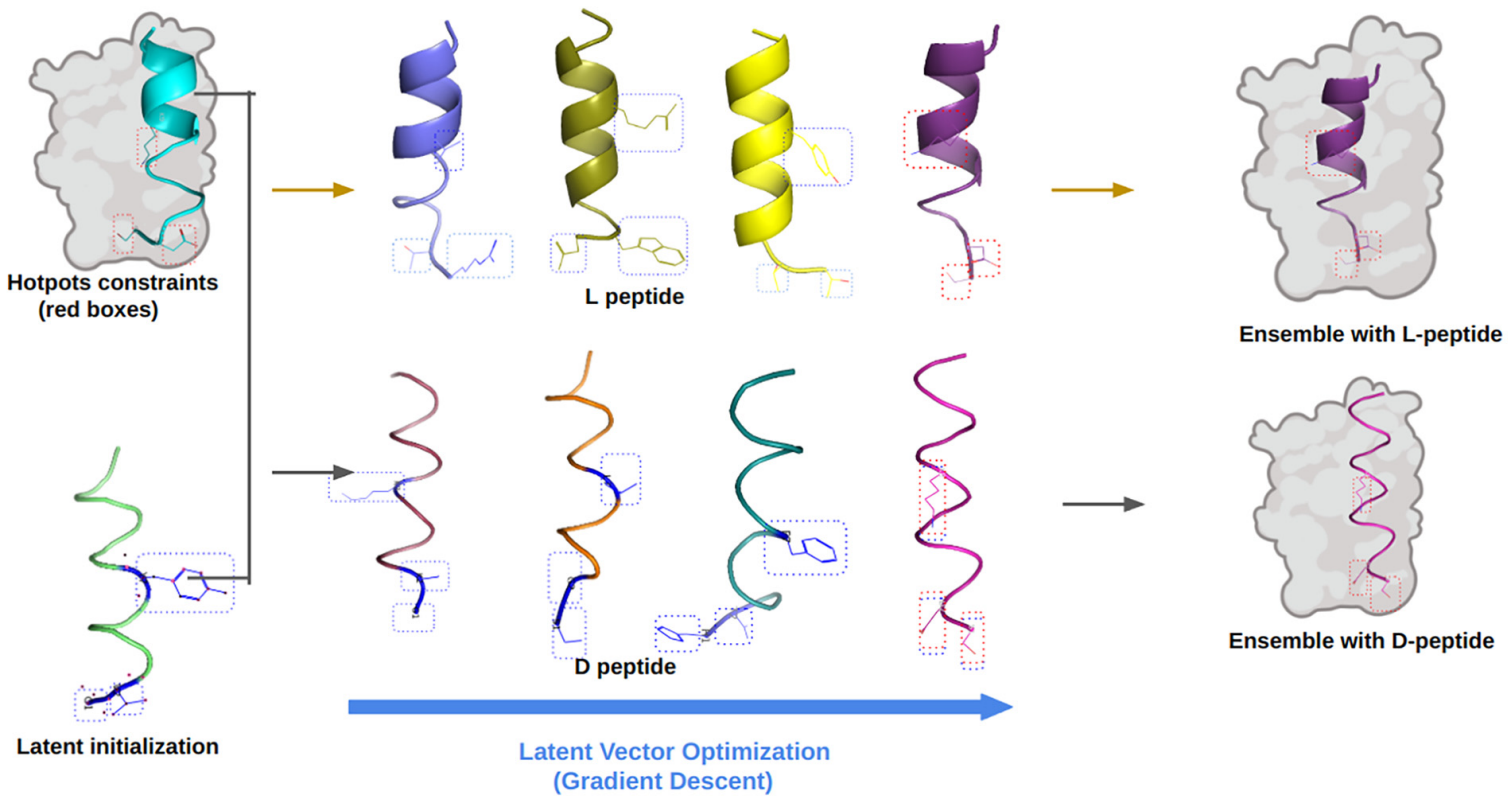
Flowchart of the *de novo* design methodology to generate peptides with constrained hotpots using HelixGAN. Design of full-atom helices using a constrained latent vector optimization. The HelixGAN model can generate L-helices directly or D-helices using mirror transformation. The constrained hotspots of the target optimization helices are shown in red boxes. The constrained regions of the optimization in the generated full-atom helices are shown in blue boxes. The top panel (yellow arrows) illustrates the constrained generation of L-peptides where the generated L-residues become closer and match the targets. The bottom panel (black arrows) shows the constrained generation of D-peptides where the generated D-residues match the targets

Hotspot residues have a significant contribution for target recognition, binding and receptor activation. Here, we constrained the structural generation of the novel peptides to a set of identified hotspots to produce functional designs. We developed a gradient-based search mechanism in latent space to optimize the generation of novel structures targeting desired hotspots. We used multiple random initial search points to decrease the probability that the system will be trapped in a local minimum during the helix generation process. Figure 4 shows *de novo* design process of peptides targeting constrained hotspots residues implemented in HelixGAN. Significantly, HelixGAN can generate L or D-peptides targeting constrained hotspots.

**Fig. 5.**
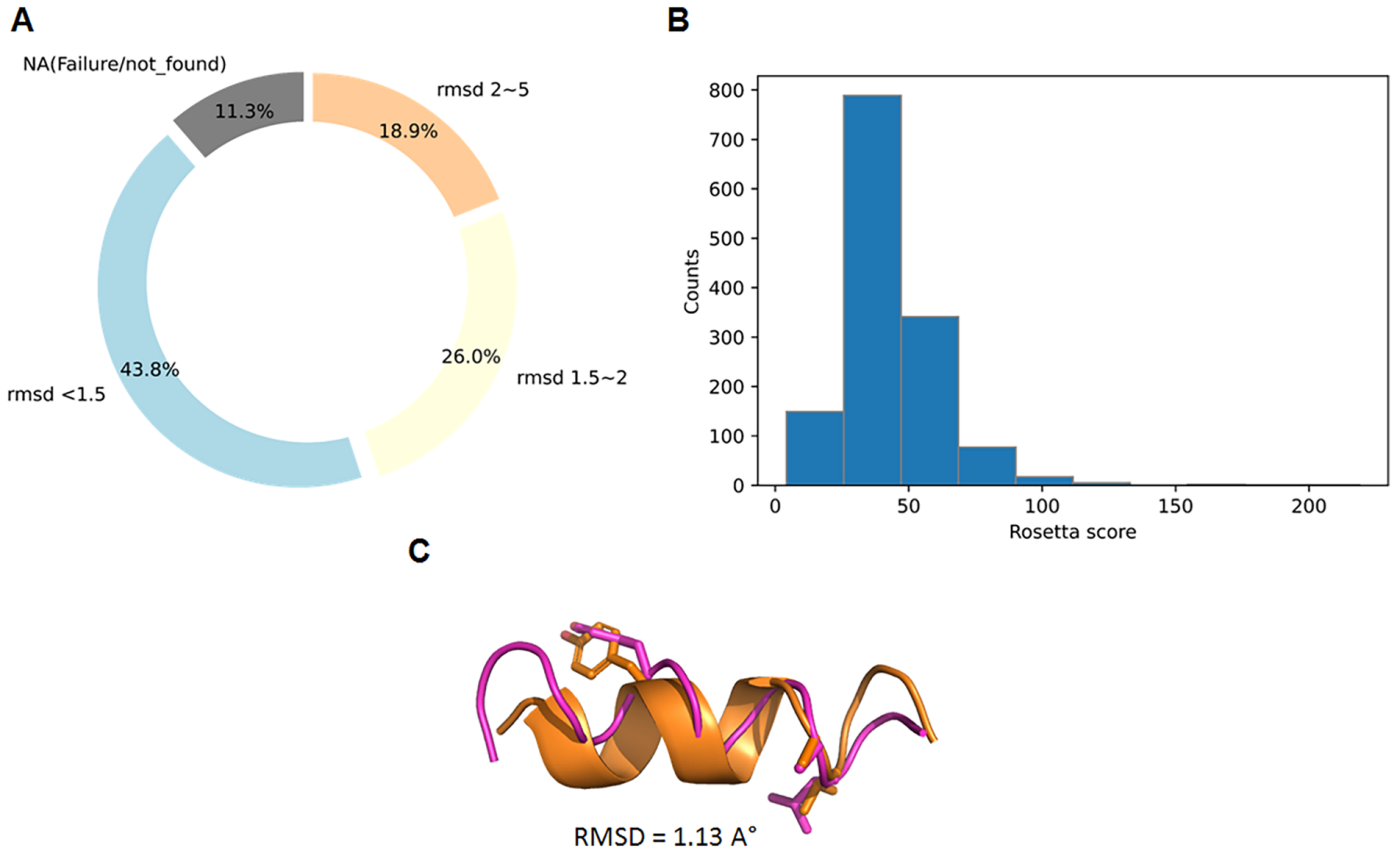
*De novo* design of L-helical peptides with constrained hotspots using the gradient search method implemented in HelixGAN. (**A**) Assessment of the performance of HelixGAN to generate L-helical peptides with constrained hotspots in a test set of 1559 samples. The RMSD was calculated between the matched and hotspots atoms in each generated and target helix. (**B**) Rosetta score distribution across the novel generated helices. (**C**) Structural alignment of one example of the target and generated helices. The target helix is shown in dark orange, while the generated helix is displayed in pink. In both helices, the match and hotspot residues are shown as licorice. The RMSD value between the aligned structures was used to evaluate the match quality

Next, we evaluated our gradient search mechanism with a trained WGAN model on the prepared test set to evaluate the performance of generating novel helices matching desired target hotspot residues. Our model generates the synthetic helix using the gradient search mechanism with a set of hotspot residues (Fig. 5A). We calculated the RMSD between the target and generated helix structures through the partial alignment between the hotspots and matched residue atoms. In 43.1% of the test cases, the RMSDs of the matched residues in the new helix structures were lower than 1.5 Å with the target hotspots. For 27% of the cases, the RMSD computed were between 1.5 and 2.5 Å (Fig. 5A). Notably, according to the Rosetta scoring function, most of the generated helix structures were classified as reasonable (Fig. 5B). We also compared HelixGAN and RosettaDesign in the generation of 100 alpha right-handed helices designs targeting three constrained hotspots. The results showed that HelixGAN outperformed RosettaDesign in the generation of L-helix peptides (Supplementary Fig. S7). Thus, our model can generate high-quality helix structures matching a given set of target hotspots for a known helix structure (Fig. 5C).

**Fig. 6.**
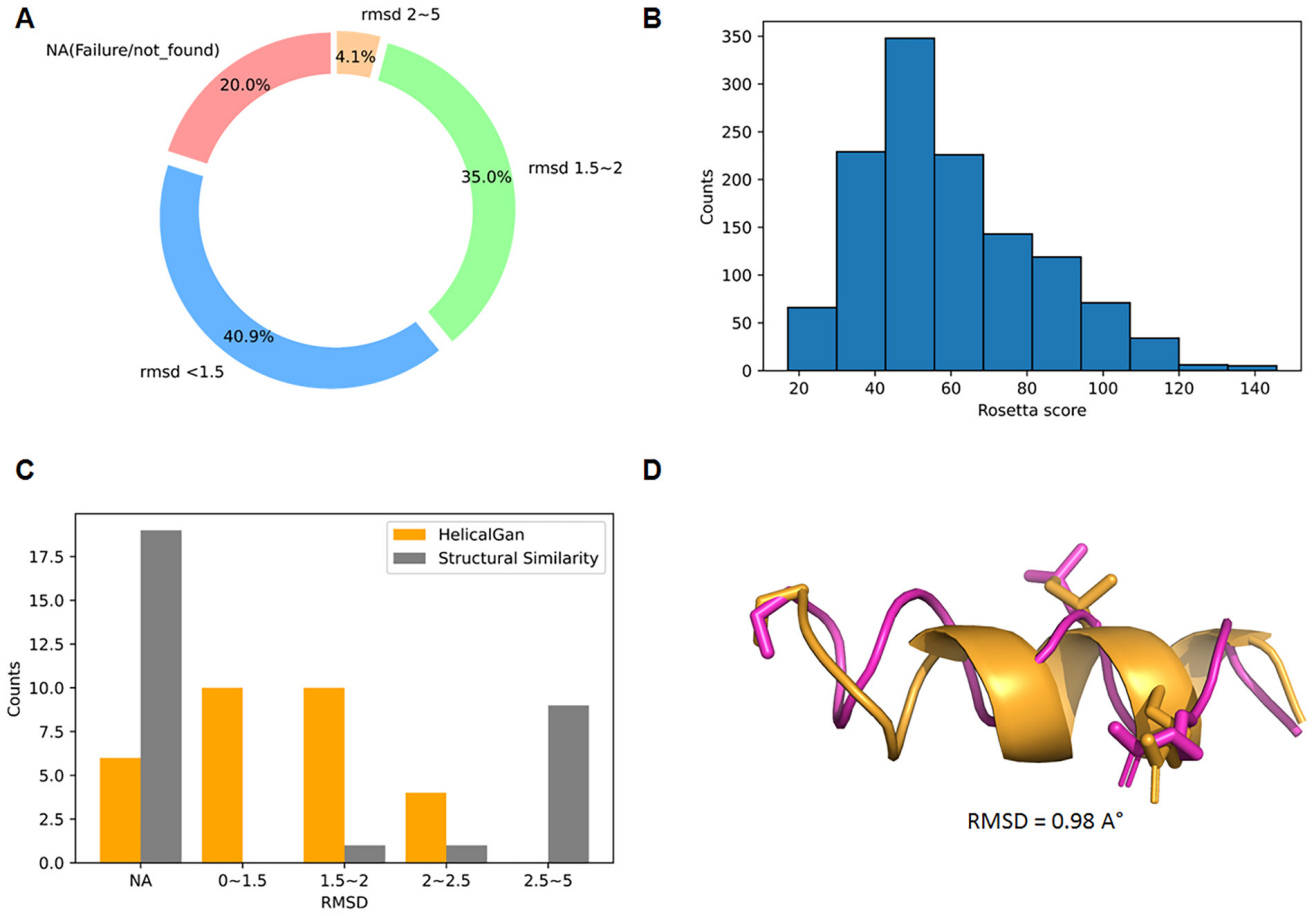
*De novo* design of D-helical peptides with constrained hotspots using the gradient search method implemented in HelixGAN. (**A**) Assessment of the performance of HelixGAN to generate D-helical peptides with constrained hotspots in a test set of 1559 samples. The RMSD was calculated between the matched and hotspots atoms in each generated and target helix. (**B**) Rosetta score distribution across the novel generated D-helices. (**C**) Comparing the performance of HelixGAN with our previous structural similarity method to generate D-helical peptides given a set of hotspots within a known L-peptide. (**D**) Structural alignment of one example of target and generated helices. The target helix is shown in dark orange, while the generated helix is displayed in pink. In both helices, the match and hotspot residues are shown as licorice. The RMSD value between the aligned structures was used to evaluate the match quality

We applied a mirror conversion as an additional step to transform the generated helices with HelixGAN into D-peptides. Given a set of hotspots in a known L-peptide, our model generates a novel D-helix peptide structure using a gradient search mechanism (Fig. 6A). Remarkably, the RMSDs of the matching residues were lower than 1.5 Å in 40.9% of the test cases. For 35% of the test cases, the RMSDs were between 1.5 and 2.0 Å (Fig. 6A). Of relevance, most of the novel D-helix structures were classified as reasonable structures based on their Rosetta scores (Fig. 6B).

We randomly selected three hotspots in 30 test cases to compare the performance of HelixGAN with our previous methodology for converting L-peptides to D-peptide analogs. The structural similarity of the D-peptides design using HelixGAN outperformed the one obtained with our previous method. HelixGAN designed D-peptide analogs with RMSD values below 1.5 Å for 10 test cases, while for other 10 cases, RMSD values in the range 1.5 2.0 Å were obtained. In contrast, our previous method found D-analogs with RMSD values in the range of 1.5–2.0 Å for only one test case (Fig. 6C). Following that, we also compared HelixGAN and RosettaDesign in the generation of 100 alpha left-handed helices designs targeting three constrained hotspots. The results showed that HelixGAN outperformed RosettaDesign in the generation of D-helix peptides (Supplementary Fig. S7). To sum up, our model can generate high-quality D-helix structures matching a given set of target hotspots for a known helix structure (Fig. 5D).

HelixGAN generates alpha-helical peptides with constrained hotspots easier for some types of residues due to different factors, such as the constraints imposed on the design of the helical peptide and the complexity (Supplementary Fig. S8). Helical peptides with more complex hotspots may require more fine-tuning parameters to generate successful designs. Other constraints imposed on the helix structure, such as the length and conformation, can influence the design’s success. Furthermore, the nature of the amino acids can also affect the design’s success, as some amino acids may be better suited for binding to specific targets than others. Further work will be necessary to understand better the underlying principles that govern the success of this approach, as well as to form strategies to better tune the GAN for targeting and optimizing specific peptide sequences.

### 3.3 *De novo* design of a D-peptide analog of GLP-1 using HelixGAN

**Fig. 7.**
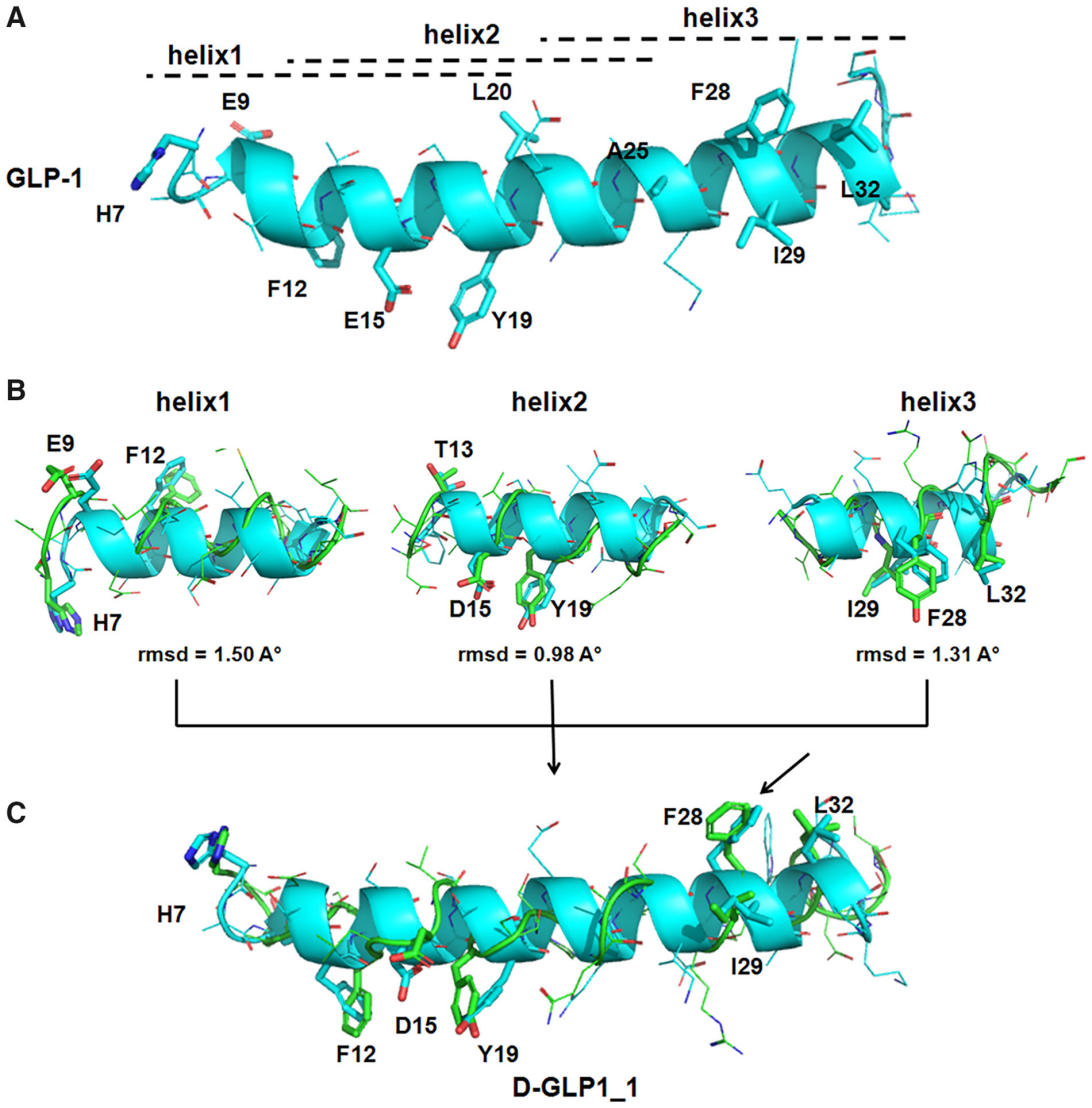
Design strategy of the novel D-GLP1_1 analog using HelixGAN. (**A**) Critical hotspots for the GLP1 function are shown in the 3D structure of the peptide as licorice. The GLP1 structure was divided into three overlapping peptides named helix1, helix2 and helix3 to design a novel D-peptide analog using HelixGAN. Helix1 extends from H7 to L20, helix2 runs from F12 to A25 while helix3 extends from Q23 to R36. The black dotted lines highlighted the extension of each helix fragment in the GLP1 structure. (**B**) Structural alignment of the best D-peptide fragments (green) obtained for helix1, helix2 and helix3 (cyan) using HelixGAN. We showed as licorice the hotspot and matching residues in the L and D-peptides. We measured the match quality through the RMSD of specific atoms. (**C**) Structural superposition of the novel D-GLP1_1 analog (green) over the GLP1 structure (cyan). We showed as licorice the hotspots and matching residues in the L and D-peptides. The black arrow indicates the mutation of Y7 in the D-peptide generated for helix3 to match better the F28 in GLP1

Here, we targeted a set of well-known hotspot residues of GLP-1 to design a novel GLP-1 D-peptide analog using HelixGAN. To generate a D-GLP1 analog, we used the ligand structure in complex with the full-length GLP-1 receptor as a starting point (PDB code: 5vai). Before designing the D-peptide analog, we divided the GLP-1 structure into three overlapping fragments named helix1, helix2 and helix3. Helix1 extends from H7 to L20, helix2 runs from F12 to A25, while helix3 runs from Q23 to R36. Positions H7, E9 and F12 were designated as hotspots in helix1. For helix2, T13, D15 and Y19 were selected as hotspots, while F28, I29 and L32 were chosen in helix3 (Fig. 7A). Previous computational and experimental studies supported our hotspot residues selection (Garton *et al.*, 2018; Manandhar and Ahn, 2015). Several D-helix structures were generated after running HelixGAN for each helix independently. The root mean square deviation (RMSD) between the specific atoms in each starting helix structure and the generated D-helix structures was used to evaluate match quality. Supplementary Figure S9 depicts the RMSD distribution profile of the best output structures from the HelixGAN search for each helix. The best match for helix1, helix2 and helix3 was found at 1.50, 0.98 and 1.31 Å, respectively (Fig. 7B). We mutated Y7 in the D-peptide fragment generated for helix3 to match better F28 in GLP1. Next, we joined the best matched peptides to generate D-GLP1_1. The structural superimposition of the resulting D-peptide design over the GLP1 structure is shown in Figure 7C. We also run MD simulations of 200 ns to evaluate the conformational stability of D-GLP1_1 and L-GLP1 (control) in solution. The simulations showed both peptides partially kept the helical secondary structure in solution (Supplementary Fig. S10). Our results fit well with the N-terminal segment unfolding observed in the NMR structure of L-GLP1 in solution (Neidigh *et al.*, 2001).

**Fig. 8.**
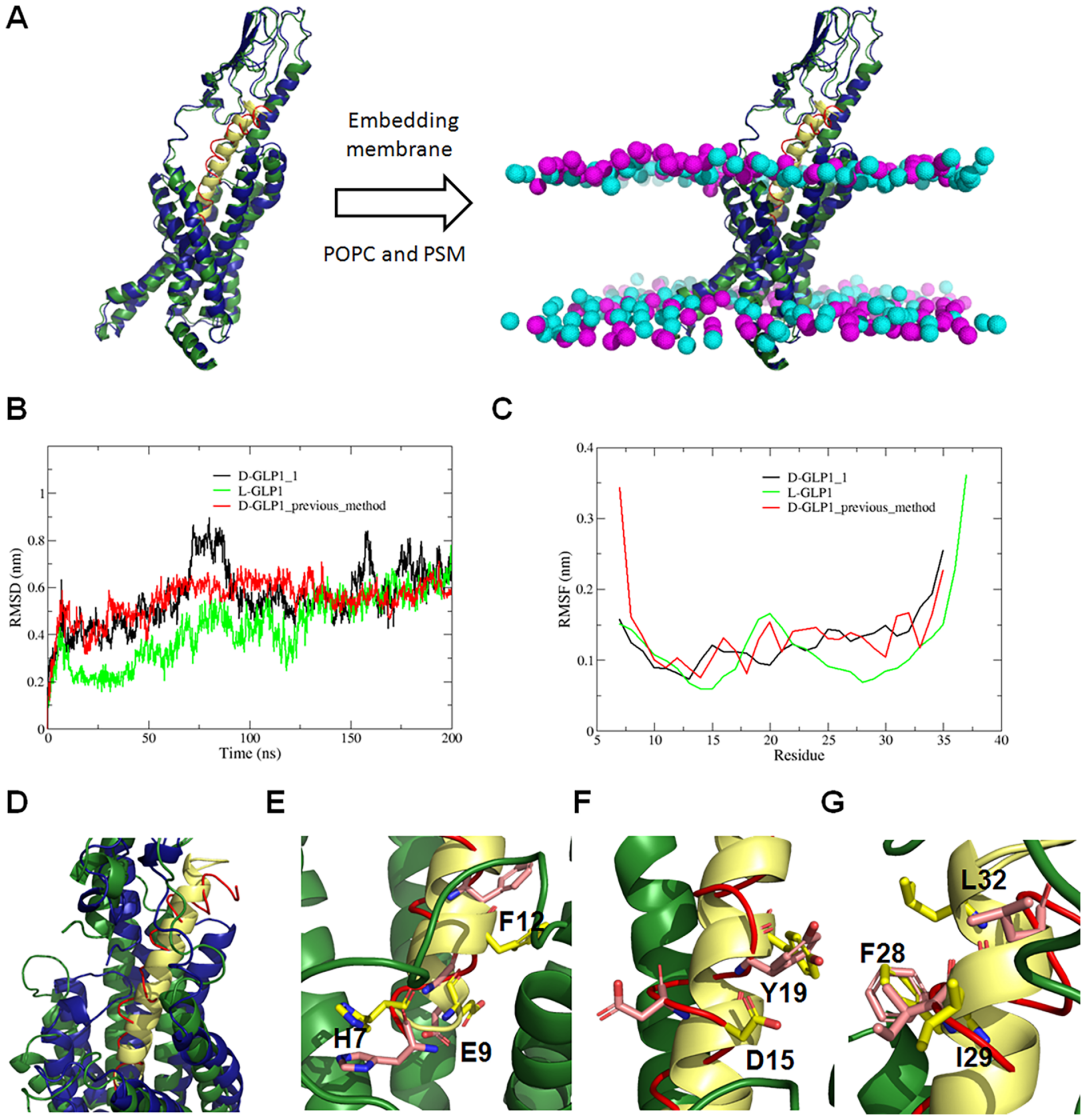
Modeling the 3D structure of the novel D-GLP1_1 analog bound to the GLP1R. (**A**) Structural superposition of the novel D-GLP1_1 analog (red) over the L-GLP1 (yellow) structure bound to the GLP1R (pdb code: 5vai). In dark green is shown the GLP1R coupled to GLP1, while in dark blue is represented the receptor bound to the D-peptide analog. (**B**) RMSD of the heavy atoms of GLP1 and two different D-peptide analogs bound to the GLP1R. The novel D-GLP1_1 analog designed in this study is displayed in black. The D-GLP1 analog created in our previous study is shown in red. The wild-type GLP1 is shown in green. (**C**) RMSF per residue of the heavy atoms of GLP1 and two different D-peptide analogs bound to the GLP1R. The D-GLP1 analog created in our previous study is shown in red. The wild-type GLP1 is shown in green. (**D**) Structural superposition of the most representative cluster extracted from the MD simulation of GLP1R+D-GLP1_1 complex over the experimental structure of GLPR1+GLP1 complex (5vai). The D-GLP1_1 analog is shown in red, while GLP1 is displayed in yellow. In dark green is shown the GLP1R coupled to GLP1, while in dark blue is represented the receptor bound to the D-peptide analog. (**E**) Zoom of the structural alignment of D-GLP1_1 (red) with GLP1 (yellow) at helix1. We showed as licorice the hotspots and matching residues (red) in the L (yellow) and D-peptides (red), respectively. (**F**) Zoom of the structural alignment of D-GLP1_1 (red) with GLP1 (yellow) at helix2. We showed as licorice the hotspots and matching residues (red) in the L (yellow) and D-peptides (red), respectively. (**G**) Zoom of the structural alignment of D-GLP1_1 (red) with GLP1 (yellow) at helix3. We showed as licorice the hotspots and matching residues (red) in the L (yellow) and D-peptides (red), respectively. In the hotspots and matching residues, the nitrogen and oxygen atoms were colored in blue and red, respectively

By superimposing the D-GLP1_1 structure onto the Cryo_EM structure of GLP1R bound to GLP1, we built the 3D structure of the GLP1R in complex with the novel D-peptide analog. The GLP1R+D-peptide complex was then embedded in a POPC: PSM (1:1) bilayer before evaluating its binding mode stability using 200 ns MD simulations (Fig. 8A and B). We also simulated the GLPR1 bound to the wild-type L-GLP1 and a D-GLP1 peptide designed earlier using our previous methodology for converting L-peptides to D-analogs as controls. The peptide designed in the current work (D-GLP1_1) shares the same orientation with the L-peptide, while the D-peptide designed previously (D-GLP1_previous_method) is retro-inverted compared to the L-peptide. This difference in the direction means that D-GLP1_1 has its N-terminal residue embedded in the transmembrane domain of the GLP1R, while the former peptide has its N-terminal residue interacting with the extracellular domain of the receptor. Similar to the L-GLP1, both D-peptides quickly stabilized in a new equilibrium position close to the initial structure, according to the RMSD profiles calculated for the peptide’s heavy atoms (Fig. 8B). The calculated root mean square fluctuation (RMSF) profiles revealed that the D-GLP1_1 analog designed with HelixGAN was more stable than the one built using our previous methodology (Fig. 8C). The first 10 residues of D-GLP1_1 mimic the structural dynamics of a similar region in GLP1 better than other peptide segments (Fig. 8C). The structural superposition of the most representative cluster extracted from the MD simulation of the GLPR1+D-GLP1_1 complex onto the x-ray structure of GLP1R bound to GLP1 revealed a significant match between specific residues in the D-peptide analog and the majority of the hotspots defined in GLP1 (Fig. 8D–G).

## 4 Discussion

Here, we presented HelixGAN, the first GAN method for designing novel helical structures from scratch at an atomic level. Unlike previous GAN models primarily focused on sequence generation (Gupta and Zou, 2018; Repecka *et al.*, 2021), our methodology directly generates sequence and structural features that mimic patterns found in real helices. HelixGAN was evaluated by comparing the distribution of the generated data’s quality and other significant parameters (sequence identity, 3D coordinates, phi, psi and chi angles) with training datasets. The performance of our model in our test samples demonstrated that it could generate physically reasonable novel helices. Implementing an algorithm to measure the duality gap of HelixGAN designs would be one of our future works. The duality gap can be used to assess the differences between the data distribution of real and generated sequences. This metric would help measure the diversity of the generated sequences (Grnarova *et al.*, 2019).

Hotspot residues play a critical role in molecular recognition, receptor activation and drug discovery (Cukuroglu *et al.*, 2014). Here, we developed a gradient-based search methodology in latent space to optimize the generation of novel helical structures targeting desired hotspots of a given functional peptide with HelixGAN. We used a separate test dataset in which we randomly selected hotspot residues in L-peptides to see if our model with a gradient search mechanism could generate helical structures matching hotspot residues conformations. Notably, HelixGAN can build novel helices that match L-type hotspot residues. However, native *α*-helical peptides are poor drug candidates due to their low conformational stability in the absence of the protein scaffold and their high sensitivity to proteolysis (Bruno *et al.*, 2013).

D-peptide analogs of bioactive peptides have clear therapeutic benefits, including low immunogenicity, low cost and increased protease stability (Garton *et al.*, 2018; Rabideau and Pentelute, 2015; Uppalapati *et al.*, 2016; Valiente *et al.*, 2021). HelixGAN outperformed our previously developed structural similarity method to generate D-peptides matching a set of given hotspots in a known L-peptide. This finding is relevant for expanding the structural space for designing D-peptides from scratch surmounting the current limited conformational space of helix structures deposited at the PDB. Next, we used HelixGAN to design a novel D-peptide analog that matches the conformations of critical hotspots for the GLP1 function. We chose GLP1 as a study case because it is currently being investigated as a diabetes mellitus and obesity treatment (Prasad-Reddy and Isaacs, 2015; Sonne *et al.*, 2014). GLP-1 is a helical GPCR agonist with several hotspots residues with distinct chemical–physical properties (Donnelly, 2012). Previous computational and experimental studies supported our hotspot residues selection (Garton *et al.*, 2018; Manandhar and Ahn, 2015). MD simulations revealed a stable binding mode of the D-GLP1_1 analog coupled to the GLP1R. Significantly, this novel D-peptide analog is more stable than our previous D-GLP1 design along the MD simulations. The differences in the orientation provoke the difference in the RMSF profile. In addition, placing the peptide in the same direction as the L-peptide should make the peptide more active because we can mimic easier the N-terminal region of the L-peptide. In the D-retro-inverted peptides, we need to modify the C-terminal moiety with an NH2 group to mimic the NH3+ partially. The importance of NH3+ moiety in activating the GPCR receptors is well-known (Gallwitz *et al.*, 2000). This increased stability would be critical for improving the peptide’s ability to activate the GLP1R. Further experiments will be needed in the future to confirm the GLPR1 activation by D-GLP1_1.

HelixGAN can generate desired alpha left- and right-handed helix structures with the help of a gradient search mechanism, although the model has yet to be trained for developing specific structural properties. Of relevance, there is a significant agreement between the helix structures generated with HelixGAN and PEP-FOLD, a well-known *de novo* approach for predicting peptide structures from amino acid sequences (Lamiable *et al.*, 2016). In the future, it will be beneficial to have the ability to optimize synthetic data as a separate pipeline for desired properties without changing the generative model. This feature will allow modularizing of the model, which increases its usefulness. For instance, if our HelixGAN model does not work for some situations, we can combine other generative methods like a variational autoenconder (Eguchi *et al.*, 2022) with gradient search to achieve the same purpose. This gradient search mechanism thus allows further synthetic biology research to integrate smoothly with the enormous capabilities of GANs. The gradient search technique is also desirable precisely because it is robust and easy to implement.

## 5 Conclusions

Here, we present HelixGAN, the first GAN method for designing novel helical structures from scratch at an atomic level. HelixGAN outperformed RosettaDesign and our previously developed structural similarity method to generate D-peptides matching a set of given hotspots in a known L-peptide. There is a significant agreement between the helix structures generated with HelixGAN and PEP-FOLD, a well-known *de novo* approach for predicting peptide structures from amino acid sequences. We envision that HelixGAN would become an essential tool for designing novel bioactive peptides with desired properties in the early stages of the drug discovery process.

## Supplementary Material

btad036_Supplementary_Data

## Data Availability

The source code and data sets are available at github (https://github.com/xxiexuezhi/helix_gan).
