# Supplementary figures and images for: HelixGAN a deep-learning methodology for conditional de novo design of α-helix structures
Source: Bioinformatics. 2023 Jan 18;39(1):btad036. doi: 10.1093/bioinformatics/btad036 (PMC9887083; doi:10.1093/bioinformatics/btad036)

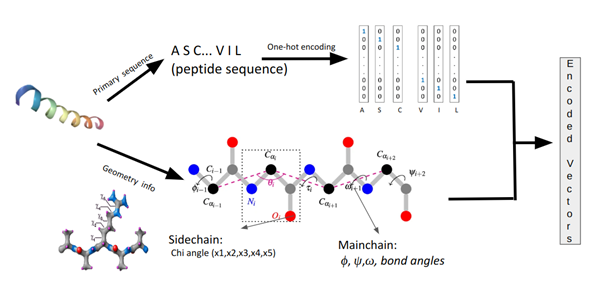

Supplement: btad036_Supplementary_Data [file btad036_supplementary_data.zip › btad036_Supplementary_Data/FigureS1.png]

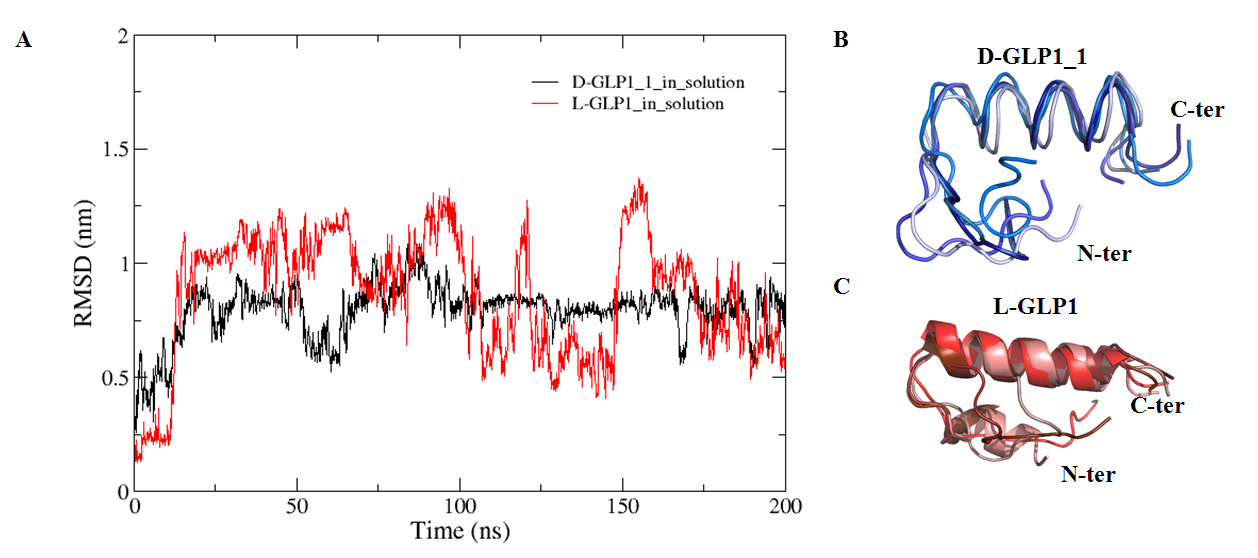

Supplement: btad036_Supplementary_Data [file btad036_supplementary_data.zip › btad036_Supplementary_Data/FigureS10.png]

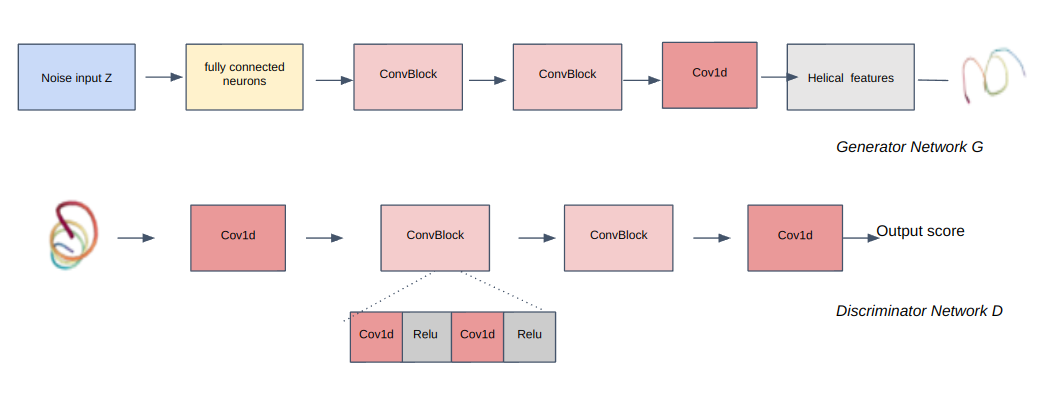

Supplement: btad036_Supplementary_Data [file btad036_supplementary_data.zip › btad036_Supplementary_Data/FigureS2.png]

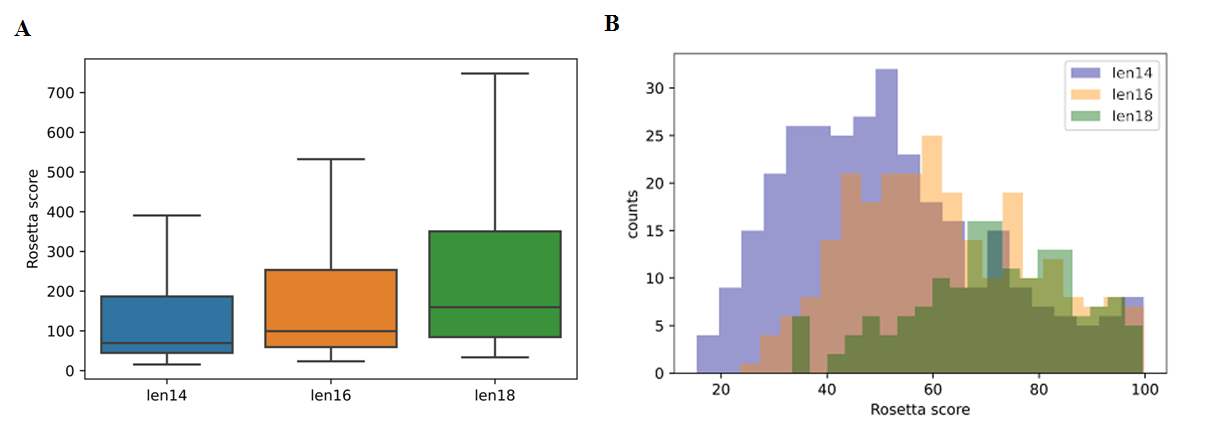

Supplement: btad036_Supplementary_Data [file btad036_supplementary_data.zip › btad036_Supplementary_Data/FigureS3.png]

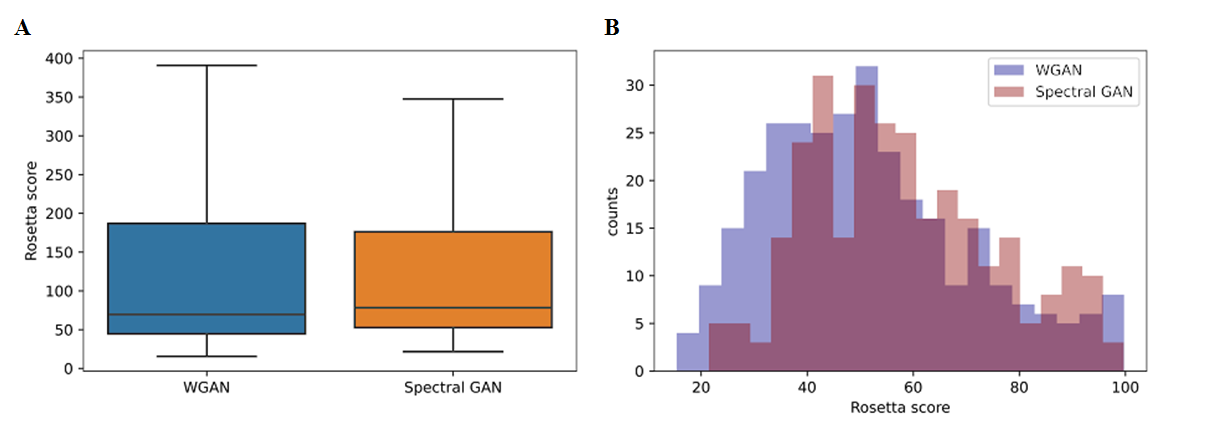

Supplement: btad036_Supplementary_Data [file btad036_supplementary_data.zip › btad036_Supplementary_Data/FigureS4.png]

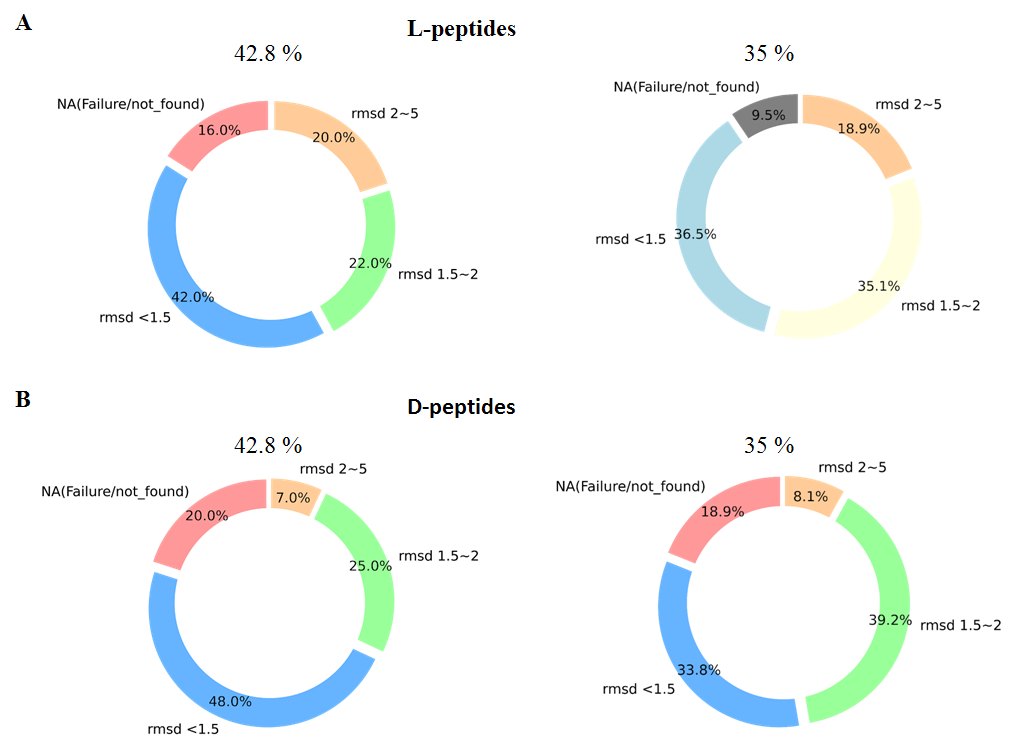

Supplement: btad036_Supplementary_Data [file btad036_supplementary_data.zip › btad036_Supplementary_Data/FigureS5.png]

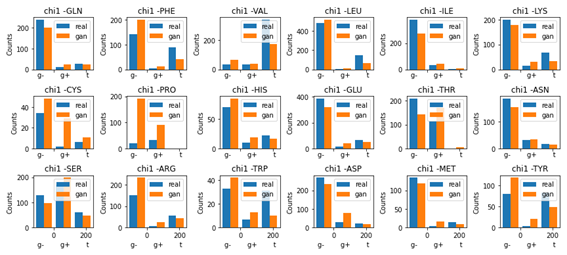

Supplement: btad036_Supplementary_Data [file btad036_supplementary_data.zip › btad036_Supplementary_Data/FigureS6.png]

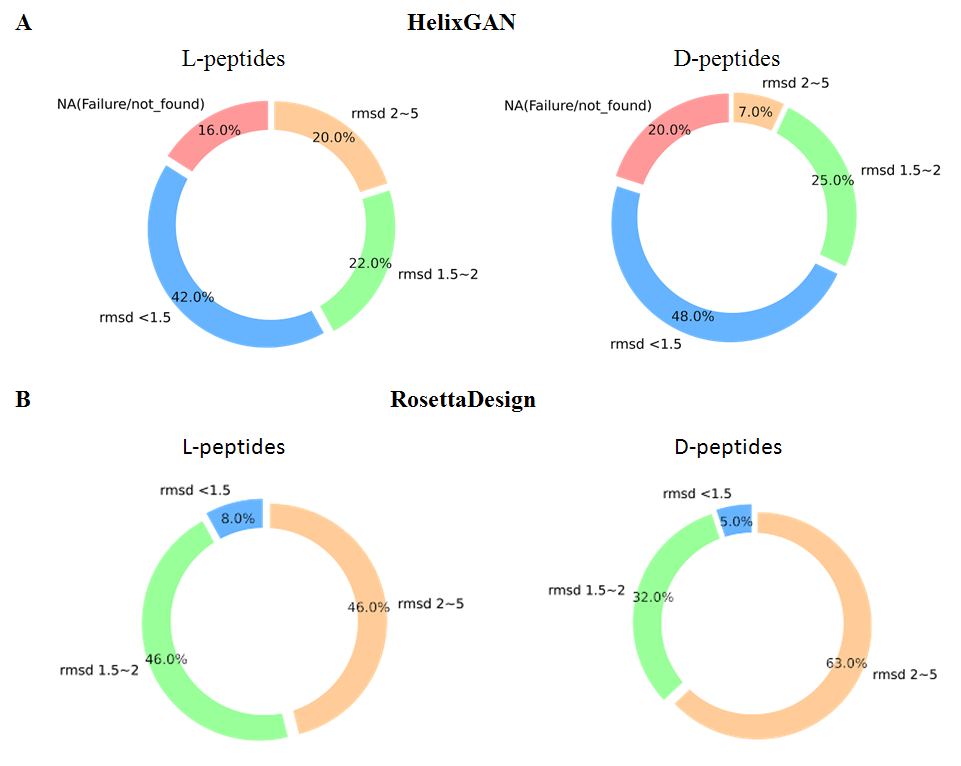

Supplement: btad036_Supplementary_Data [file btad036_supplementary_data.zip › btad036_Supplementary_Data/FigureS7.png]

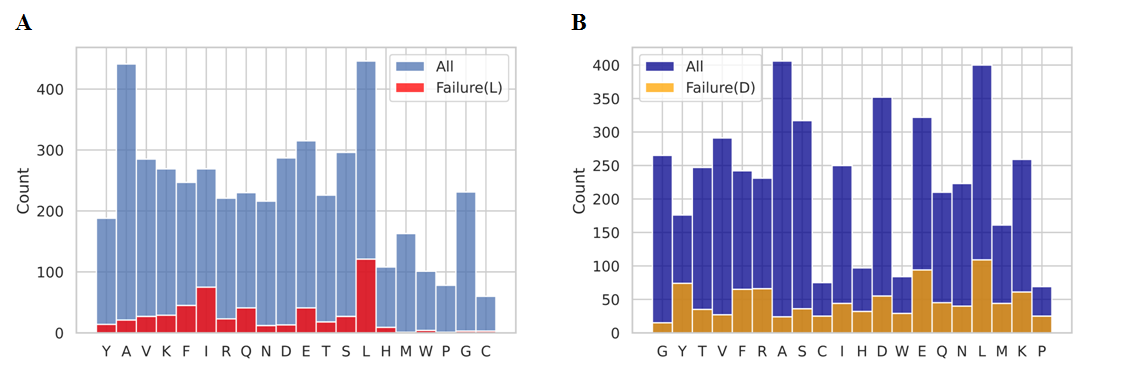

Supplement: btad036_Supplementary_Data [file btad036_supplementary_data.zip › btad036_Supplementary_Data/FigureS8.png]
